# Phosphomannomutase 2 hyperinsulinemia: Recent advances of genetic pathogenesis, diagnosis, and management

**DOI:** 10.3389/fendo.2022.1102307

**Published:** 2023-01-16

**Authors:** Congli Chen, Yanmei Sang

**Affiliations:** Department of Pediatric Endocrinology, Genetic and Metabolism, Beijing Children’s Hospital, Capital Medical University, National Center for Children’s Health, Beijing, China

**Keywords:** congenital disorder of glycosylation, diazoxide, congenital hyperinsulinism, hypoglycemia, phosphomannomutase 2

## Abstract

Congenital hyperinsulinemia (CHI), is a clinically heterogeneous disorder that presents as a major cause of persistent and recurrent hypoglycemia during infancy and childhood. There are 16 subtypes of CHI-related genes. Phosphomannomutase 2 hyperinsulinemia (PMM2-HI) is an extremely rare subtype which is first reported in 2017, with only 18 families reported so far. This review provides a structured description of the genetic pathogenesis, and current diagnostic and therapeutic advances of PMM2-HI to increase clinicians’ awareness of PMM2-HI.

## Search strategy and selection criteria

We searched PubMed for full-text original studies and case reports written in English, to identify reports about the genetic pathogenesis, diagnosis, and management pathophysiology, consequences, and treatment of phosphomannomutase 2 hyperinsulinemia. The search terms used were “hyperinsulinism”, “congenital hyperinsulinism”, “congenital hypoglycemia”, “phosphomannomutase 2”, and “PMM2 protein”.

The reference lists of the identified papers were also used to identify further papers of interest. The final reference list was selected on the basis of relevance to this study.

## Introduction

Congenital hyperinsulinism (CHI) is one of the principal causes of persistent, recurrent hypoglycemia in infancy and childhood, and is highly genetically and clinically heterogeneous. At least 16 CHI-related pathogenic genes (ABCC8, KCNJ11, GLUD1, GCK, HADH, SLC16A1, UCP2, HNF4A, HNF1A, HK1, KCNQ1, CACNA1D, FOXA2, EIF2S3, PGM1, and PMM2) have been identified so far and are involved in the regulation of insulin secretion from pancreatic β-cells (1, 2). Nevertheless, almost 40% of children with CHI still have unidentified genes to date (3). Among the clinical subtypes, adenosine triphosphate-sensitive potassium channel hyperinsulinism (KATP-HI) is the most common and most clinically severe subtype, accounting for approximately 40-50% of CHI patients. Glutamate dehydrogenase hyperinsulinism (GDH-HI) is the second most common type, and the remaining subtypes are extremely rare. Zinc figure protein 143 (ZNF143) has an altered affinity for phosphomannomutase 2 (PMM2) gene promoter due to a variant in the promoter of the PMM2 gene, resulting in abnormal expression of the PMM2, which is a principal N-glycosylation enzyme encoded by the PMM2 gene in the pancreas. Consequently, this results in abnormal insulin secretion from pancreatic β-cells and phosphomannomutase 2 hyperinsulinism (PMM2-HI).

PMM2-HI is one of the extremely rare types of CHI, with approximately at least 18 families and over 26 patients with PMM2-HI reported to date. This review will illustrate the recent advances in genetic pathogenesis, diagnosis, and management regarding PMM2-HI, in an attempt to contribute to clinicians’ awareness of the disease, which in turn will facilitate the early diagnosis and management of hypoglycemia in infancy and childhood.

## PMM2-HI

In 2004, Müller et al. reported a 5-year-old patient with polycystic kidney disease accompanied by hyperinsulinemic hypoglycemia, and the first clinical association between polycystic kidney disease with hyperinsulinemia was described, but the genetic pathogenesis was not further elucidated (4). In 2007, Cabezas et al. (5) described the association of PMM2 gene promoter variants with hyperinsulinemic hypoglycemia (HH) and autosomal recessive polycystic kidney disease (ARPKD), first proposing the concept of PMM2-HI. Using Sanger sequencing of 17 children with concurrent HH and ARPKD from 11 unrelated European pedigree families, Cabezas et al. identified promoter (c.-167G>T) pure or trans-coding mutations in the PMM2 gene of all patients, with the four patients from the consanguineous families having a pure heterozygous status and the others with a compound heterozygous status. These patients did not present with the characteristic clinical features and laboratory findings of phosphomannomutase-2-congenital disorders of glycosylation (PMM2-CDG, OMIM: #212065), which are the most common disease with variants in the PMM2 but presenting only with symptoms of ARPKD, hepatocytes, and hypoglycemia. Most of these patients present at birth as giant fetuses. The median onset of hypoglycemia in the children was 10 months of age, with only one patient with onset at 4 years of age and the rest within 1 year of age, typically presenting with seizures. Most patients are responding effectively to diazoxide treatment, with a small number of patients spontaneously resolving without treatment (5). Since then, several researchers have identified and supplemented the nucleotide sequences of PMM2 gene promoter mutations in patients diagnosed with HH and ARPKD (6–10). In 2020, Moreno et al. illustrated six patients in four unrelated Spanish families with variants in the PMM2 gene and present with PMM2-HI and ARPKD, all of whom had the heterozygous variant c-167G>T in the PMM2 promoter region. Moreno et al. presented that patients with PMM2 promoter mutations might also carry PMM2-CDG-associated mutations, but only 2 patients suffered from the PMM2-CDG phenotype (7).

PMM2-CDG is a disease that was first proposed in 1997 (11) and can present hyperinsulinemia, but most of the currently known literature on PMM2-CDG only mentions the hypoglycemic phenotype and insulin level at the time of hypoglycemia was not demonstrated detailed (12).

## PMM2 gene and PMM2 protein

**Figure 1:**
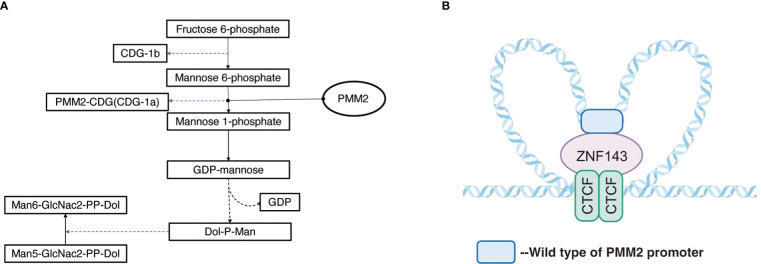
**(A)** Pathway of PMM2 involved. Mannose-1-phosphate is a precursor of guanosine diphosphate (GDP-mannose) which is a material required for the synthesis of polyphenol-P-oligosaccharide (Dol-P-Man) and is further involved in the synthesis of mannose and protein glycosylation. **(B)** Regulation mechanism of PMM2 promoter: ZNF143 interacts with specific CTCF sites, and by connecting CTCF with the remote regulatory elements, it affects the formation of chromatin loops, carrying out specific gene regulation.

The PMM2 gene, located on chromosome 16p13, is a small gene consisting of eight exons with a total length of approximately 6.7kb (https://www.ncbi.nlm.nih.gov/gene/5373). PMM2 is a protein consisting of 246 amino acids (11) and is widely expressed in the digestive tract, lymph nodes, adrenal glands, adipose tissue, pancreas, liver, brain tissue, and other tissues, with the highest concentrations observed in the colon and duodenum PMM2. PMM2 catalyzes the initiation of the N-glycosylation process and the second step of the mannose pathway, the isomerization of mannose-6-phosphate to mannose-1-phosphate, in the metabolism of fructose and mannose, amino and Nucleotide glycans in humans. Mannose-1-phosphate as a precursor of guanosine diphosphate (GDP-mannose), is a material required for the synthesis of polyphenol-P-oligosaccharide (Dol-P-Man), which is further involved in the synthesis of mannose and protein glycosylation (**Figure 1**) **(** 13). In particular, variants in the PMM2 gene have been proved to contribute to defects in the protein glycosylation pathway (11, 13–16), manifesting as carbohydrate-deficient glycoprotein syndrome type I (congenital disorders of glycosylation, type 1a, CDG1a), with CDG1a the most common CDG disorder. The sulfonylurea receptor (SUR) is a key protein in the regulation of insulin secretion, and it was demonstrated that glycosylation plays an important role in SUR receptor-targeted membrane transportation (17), and Cabezas et al. further revealed *in vitro* experiments that insulin secretion from pancreatic β-cells was markedly changed after SUR deglycosylation, with consequent severe effects on human metabolism.

## Mutation profiles of PMM2-HI-related genes

**Figure 2:**
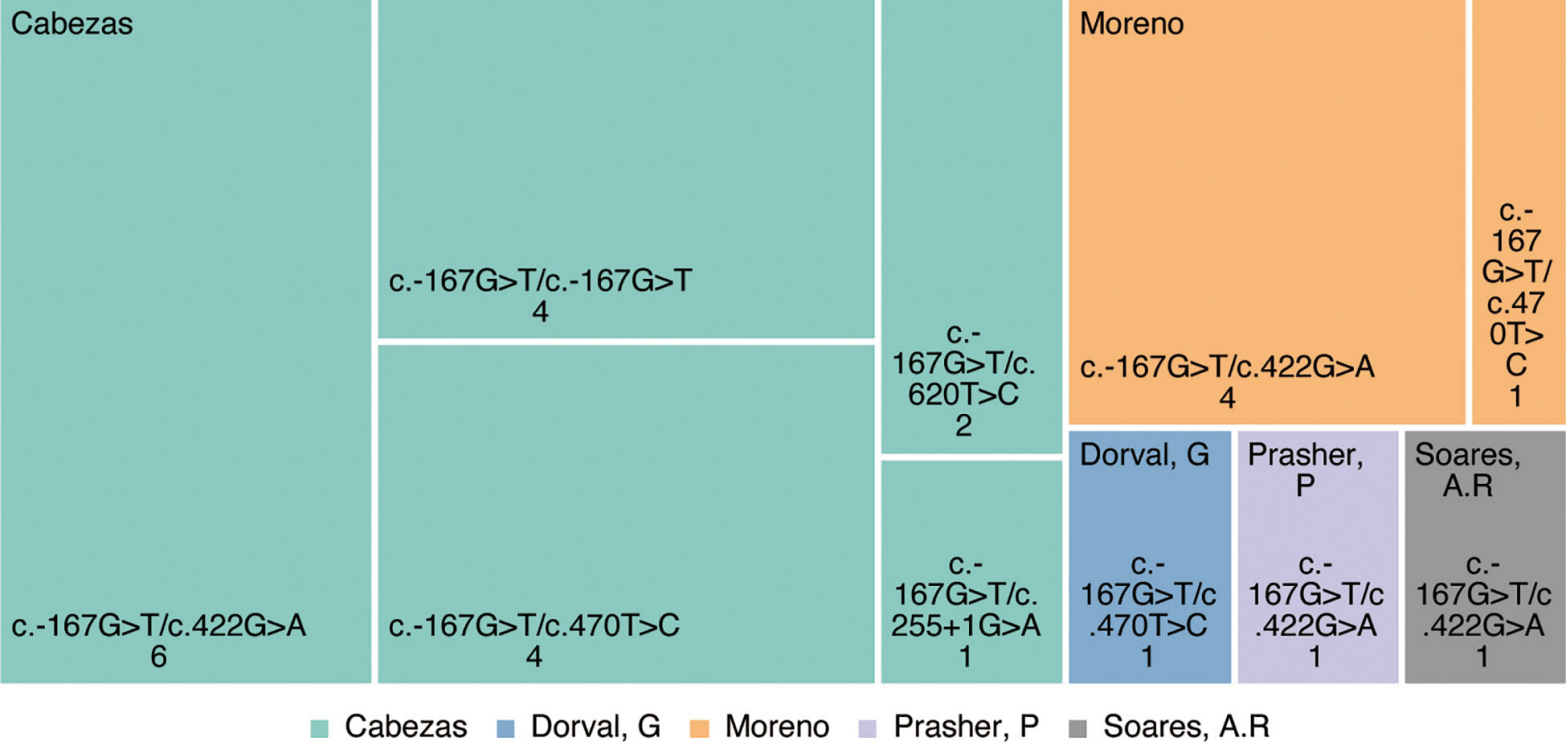
Variants in patients with PMM2-HI reported in the literature.

Cumulatively, 144 mutation types were reported in PMM2 retrieved from the Human Gene Mutation Database (HGMD, www.hgmd.cf.ac.uk). As research progressed from Cabezas (5) to date, PMM2-HI has been reported in at east 18 families with more than 26 patients with PMM2-HI, and all were inherited as autosomal compound heterozygous recessive genes. At least five variant types have been reported, including four missense mutations and one splice variant (**Figure 2**). In each of these patients, the c.-167G>T variant, which has been proven to be a PMM2 promoter variant, leads to a decreased affinity for the PMM2 promoter ZNF143 (5), which contributes to defective glycosylation in the kidney and pancreas by altering the forming of the tridimensional structure of the chromatin ring and thus tissue-specific modulation of PMM2 enzyme transcription *in vivo*. Additionally, the ClinVar and OMIM databases were searched that the four remaining PMM2 gene variant types (c.422G>A(p.Arg141His), c.470T>C(p.Phe157Ser), c.620T>C (p.Phe207Ser), and c.255+1G>A) have all been previously described in patients with different types of CDG1a syndrome.

## Genetic pathogenesis of PMM2-HI

Previous studies have shown (18) that the tridimensional structure of chromatin plays a key role in the regulation of gene expression in the coding region according to noncoding regions of genes. The loops structure, also known as topologically associated domains (TAD), is composed of chromatin and CCCTC binding factor (CTCF) on both sides, acting as the basic unit of transcriptional regulation and influences the tridimensional structure of chromatin (19, 20). The area around PMM2 contains a few CTCF sites and functional promoters. Pairs of CTCF sites interact with each other to form local chromatin loops. ZNF143 interacts with specific CTCF sites (**Figure 1**), and connecting CTCF with the remote regulatory elements affects the formation of chromatin loops, carrying out specific gene regulation (20–23).

Although PMM2 is generally expressed in a variety of tissues, it was demonstrated that PMM2 expression possessed certain tissue specificity (24). Hepatocyte nuclear factor 4 alpha (HNF4A), one of the transcription factors, is mainly expressed in the liver, pancreas, and kidney, and HNF4A promotes the binding of wild-type PMM2 promoter to ZNF143 during transcription to establish a functional chromatin loops loop, while the mutual interaction of mutant PMM2 promoter with ZNF143 and CTCF interactions are disrupted by HNF4A and PMM2 enzyme transcription is decreased (18, 19, 22). Thus, PMM2 promoter variant patients manifested symptoms only in the liver, pancreas, and kidneys.

Decreased PMM2 enzyme expression in the liver, pancreas, and kidneys leads to impaired crucial glycosylation in their respective organs. As a consequence of impaired key protein glycosylation, it manifests in the liver as prenatal ultrasound or postnatal bile duct dilatation as well as congenital hepatic fibrosis; in the kidney it can be observed as bilateral renal enlargement, diffuse echogenic kidney, pathological examination revealing renal cysts (mainly of tubular origin) as well as huge renal cysts that resemble ARPKD. The mechanism of PMM2-HI relates to pancreatic SUR receptor deglycosylation, a key protein in the regulation of insulin secretion, and the evidence indicates (17) that glycosylation plays an essential role in SUR receptor-targeted membrane transport. SUR is a key protein in the modulation of insulin secretion. Cabeza et al. found (5) that after the stimulation of mouse β-cells with protein kinase C activator insulin secretion significantly increased after stimulation with protein kinase C activator, which demonstrates that deglycosylation exerts a significant effect on insulin secretion.

Pathological specimens of the pancreas in affected patients are also rare by the extremely rare clinical occurrence of PMM2-HI. Only two cases of pancreatic pathology in patients with PMM2-HI have been reported: one patient’s pancreatic histology showed mild dilatation of the pancreatic ducts, and one patient’s pancreas showed no abnormalities (10). Moreover, to the best of our acknowledgment, no 18F-L-DOPA-PET pancreatic scan results of PMM2-HI patients have been reported in the literature, which prevents definitive staging for the time being, and more research is urgently needed in the future.

## Clinical characteristics of PMM2-HI

The majority of PMM2-HI patients reported to date have an autosomal recessive mode of inheritance and are from families of European ancestry. 25% probability of CHI occurring in children born to parents with PMM2-HI children and most of these patients are overweight at birth. A few cases may have onset in childhood, with the latest reported age of onset being 4 years. No association has been found between the age of onset and the severity of symptoms.

The clinical manifestations of PMM2-HI patients are primarily polycystic kidneys, hyperinsulinemic hypoglycemia, and polycystic liver. Bilateral renal enlargement, diffuse echogenic kidneys, and diminished hepatic parenchymal differentiation, and multiple small cysts around the liver may be detected on prenatal ultrasound or after birth. Chronic kidney disease symptoms progress slowly, especially after the age of 35 years when the progression of kidney disease is further slowed.

Patients with hypoglycemia mostly onset with epilepsy, without the manifestation of high blood ammonia and liver function abnormalities, occasionally with high serum lactate. Compared with CDG1a patients with PMM2 gene variant, PMM2-HI patients showed no neurological impairment and no transferrin hydroelectric focusing. However, in a case reported by Dorval et al. (10) fetal ultrasonography showed abnormally enlarged kidneys at 23 weeks of the first pregnancy in an unrelated couple. After the termination of pregnancy at 25 weeks, neuropathological examination showed cerebellar hypoplasia with the denticulate nucleus and optic nerve defects, presumably associated with the patient’s simultaneous heterozygosity for CDG1a-related variants.

## Diagnosis of PMM2-HI

PMM2-HI is diagnosed in the same manner as other types of CHI, based on the child’s presentation of non-ketotic hypoglycemia, requiring large amounts of glucose infusion to control hypoglycemic episodes, and overproduction of insulin which is incompatible with hypoglycemia. According to the guidelines published by Ferrara et al. (25) in 2016, the diagnostic criteria for CHI are as follows: when intravenous plasma glucose <2.8 mmol/L accompanied by asynchronous insulin secretion (usually >1-2 μU/ml; or still detectable C-peptide >0.2 mmol/L), low levels of β-hydroxybutyric acid (<1.8 mmol/L), low free fatty acids (<1.7 mmol/L), positive glucagon stimulation test (test procedure: 1 mg glucagon intramuscularly or intravenously, a neonatal dose of 0.5 mg, blood glucose elevation ≥1.7 mmol/L). Besides the above indicators, PMM2-HI can also be diagnosed by genetic examination with the genetic variant c.-167G>T, which is typical differential diagnostic evidence of PMM2-HI.

## Treatment of PMM2-HI

The treatment of PMM2-HI patients in the acute stage is consistent with those of other types of CHI patients, in which rapid infusion of glucose (26) (reported in the literature (6) at a maximum rate of up to 17.6 mg/kg/min) can be infused to prevent and mitigate the onset of hypoglycemia in order to avoid permanent damaging of the neurological system of the child due to hypoglycemic episodes.

Pharmacologically, diazoxide is the first choice for the control of hypoglycemic episodes in CHI patients, and it is a KATP channel activator (27–29). Since PMM2 promoter gene variants did not induce structural changes in KATP channels, about 54% (14/26) of patients received diazoxide treatment, and it was shown that all PMM2-HI patients responded effectively to diazoxide treatment. The most frequent complications of diazoxide are sodium retention and pulmonary hypertension (30, 31). In children who are at risk of water and sodium retention and pulmonary hypertension and require high volumes of glucose infusion to control the occurrence of hypoglycemia, a thiazide diuretic (hydrochlorothiazide 1-2mg/kg/day, bid) can be used in advance to prevent heart failure (32). Other side effects of diazoxide include neutropenia, blood volume hypertension, and hypertrichosis. There is no literature reported on the application of octreotide to treat patients with PMM2-HI.

Using captopril (0.2 mg/day) is also recommended to improve renal function in patients with decreased GFR due to polycystic kidneys.

To date, there is no literature reporting the following treatments for PMM2-HI: hormone therapy (such as glucagon) and surgical therapy (focal lesion resection or diffuse lesion near-total pancreatectomy), which are commonly used as treatment strategies for patients with CHI besides pharmacological treatment.

## Prognosis of PMM2-HI

As reported in the available studies (5–10, 12), patients with PMM2-HI treated with diazoxide are clinically effective with diazoxide medication, hence, if the diagnosis and reasonable treatment are performed early, complications such as neurological damage due to persistent recurrent hypoglycemia can be effectively avoided and the clinical prognosis is generally promising. There is no report of spontaneous recovery in children with PMM2-HI.

The majority of children with PMM2-HI have an early onset of symptoms and are most effective on diazoxide therapy. In clinical practice, children with prenatal ultrasound diagnosis of polycystic kidney for which attention should be paid to monitor their blood glucose and insulin levels. Once the child’s blood glucose has recovered to normal levels, follow-up testing of the child’s kidney and liver function should be performed. Genetic tests such as sanger should be performed promptly in children with diagnosed CHI which can assist in genetic typing, formulation of a more effective and precise therapeutic strategy, and effectively improve the prognosis of children with CHI (33–35).

Meanwhile, more basic research and clinical research reports on the mechanism of PMM2 promoter variants are needed in the future to further clarify the type of pancreatic pathology, neurological damage symptoms, and therapeutic options for PMM2-HI.

## Author contributions

CC wrote the manuscript and performed the research in the medical literature. YS reviewed the manuscript. All the authors approved the final manuscript as submitted and agree to be accountable for all aspects of the work.
